# Related to testes-specific, vespid and pathogenesis protein-1 is regulated by methylation in glioblastoma

**DOI:** 10.3892/ol.2014.1829

**Published:** 2014-01-27

**Authors:** ELAD JACOBY, MICHAL YALON, MOSHE LEITNER, ZVI R. COHEN, YEHUDIT COHEN, TAMAR FISHER, SARIT EDER, NINETTE AMARIGLIO, GIDEON RECHAVI, SIMONA CAZACU, CUNLI XIANG, TOM MIKKELSEN, CHAYA BRODIE, AMOS TOREN

**Affiliations:** 1 Department of Pediatric Hematology and Oncology, Affiliated to Sackler School of Medicine, Tel Aviv University, Ramat Gan, Tel Aviv 52621, Israel; 2 Cancer Research Center, Affiliated to Sackler School of Medicine, Tel Aviv University, Ramat Gan, Tel Aviv 52621, Israel; 3 Department of Neurosurgery, The Chaim Sheba Medical Center, Affiliated to Sackler School of Medicine, Tel Aviv University, Ramat Gan, Tel Aviv 52621, Israel; 4 Davidson Laboratory of Cell Signaling and Tumorigenesis, Hermelin Brain Tumor Center, Department of Neurosurgery, Henry Ford Hospital, Detroit, MI 48202, USA; 5 The Mina and Everard Goodman Faculty of Life Sciences, Bar-Ilan University, Ramat Gan, Tel Aviv 52900, Israel

**Keywords:** glioblastoma, related to testes-specific vespid and pathogenesis protein-1, glioma pathogenesis-related protein 1, methylation

## Abstract

Related to testes-specific, vespid and pathogenesis protein-1 (RTVP-1), also known as glioma pathogenesis-related protein 1, is highly expressed and has oncogenic features in glioblastoma (GBM; World Health Organization class IV). Promoter methylation has been found to control RTVP-1 expression in prostate carcinoma, Wilms’ tumor, acute myeloid leukemia and melanoma. In this bi-institutional study, the methylation status of RTVP-1 in astrocytic brain malignancies (GBM and oligodendroglioma) was examined. The RTVP-1 promoter was hypomethylated in GBM compared with non-tumor brain samples, but was hypermethylated in oligodendroglioma. RTVP-1 methylation correlated with RTVP-1 expression at the mRNA level. In GBM, hypermethylation of the RTVP-1 promoter was associated with improved overall survival although with no statistical significance.

## Introduction

Glial tumors are the most common brain malignancies, accounting for major morbidity and mortality of adults and children. These tumors are subclassified by the World Health Organization (WHO) into four grades according to pathological appearance, mitotic activity, vascular proliferation and regional necrosis (1). Glioblastoma (GBM; WHO grade IV) is the most aggressive type of glioma, with a median survival time of 8–14 months (2). The majority of GBM tumors are considered primary tumors, whereas only 5% are secondary- to low-grade glioma, characterized by various clinical and molecular features. Despite improved understanding of the biology of glioblastoma and newly available treatment modalities, survival rates have not changed in the past decades.

DNA methylation, one of the most common epigenetic modifications, has been widely described in cancer, which exhibits global DNA hypomethylation in addition to significant hypermethylation (and thus downregulation) of tumor suppressor genes (3). Aberrant DNA methylation is well described in GBM (4,5).

Related to testes-specific, vespid and pathogenesis protein-1 (RTVP-1), or glioma pathogenesis-related protein 1, is highly expressed in GBM and glioma cell lines, but not in normal adult brain, nor in low grade astrocytomas, oligodendrogliomas or other nervous system tumors (6–9). Previous studies have reported that RTVP-1 can be epigenetically regulated (10–13), but this has not been demonstrated in central nervous system tissue or tumors.

In the current study, RTVP-1 promoter methylation status in GBM and the association of promoter methylation with disease progression and patient outcome were examined.

## Materials and methods

### Tissue and tumor samples

Tissue and tumor samples and patient data were obtained from Sheba Brain Tumor Bank (Tel Aviv, Israel) and Henry Ford Hospital (Detroit, MA, USA). Patient data was subsequently anonymised. In total, 69 tissue samples from different patients were received: 43 GBM, 16 oligodendroglioma and 10 samples of non-tumor brain (excised from epilepsy patients). All samples were from unrelated patients and none were from secondary tumor or tumor relapse. The median age of GBM patients was 57 years (range, 35–74 years) and 47 years (range, 24–81 years) for oligodendroglioma patients. Males accounted for 56% of patients. Each specimen was assigned a 3-character code for identification between the sample and the anonymous data. The specimen collection was approved by the institutional ethical committee of each center.

### DNA extraction and bisulfite treatment

DNA was extracted according to the manufacturer’s instructions of the DNeasy kit (spin-column) (Qiagen, Germantown, MD, USA). Bisulfite treatment was performed on 1 *μ*g DNA using an EZ DNA Methylation-Gold kit (Zymo Research, Orange, CA, USA) according to the Sequenom protocol (San Diego, CA, USA) (14). The final elution volume of C–T converted DNA was 50 *μ*l.

### Methylation analysis

**Figure 1:**
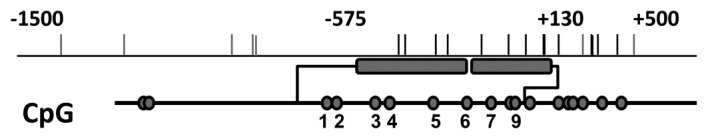
Schematic representation of related to testes-specific, vespid and pathogenesis protein-1 promoter between −1,500 bp upstream and +500 bp downstream of the transcription start site, with each CpG site marked. Grey lines indicate CpG sites that were not detectable by Sequenom, while black lines indicate sites that were detectable. The amplicons assessed are schematically marked by boxes.

Promoter sequences were searched using the University of California, Santa Cruz genome browser (http://genome.ucsc.edu). CpG sites were searched within the promoter area between −1,500 bp upstream and +500 bp downstream of the transcription start site (TSS). Methylation analysis was performed by mass spectrometry (Sequenom Bruker mass spectrometer and Sequenom Samsung MassARRAY Nano dispenser, Sequenom Inc., San Diego, CA, USA) of base-specific cleavage products, according to the Sequenom protocol previously described (14). Amplicons and primers were designed using Sequenom EpiDesigner (http://www.epidesigner.com), as presented in Fig. 1 and Table I. Polymerase chain reaction (PCR) was carried out with 50 ng bisulfite-treated DNA made up to a total volume of 25 *μ*l with GoTaq buffer (Promega, Madison, WI, USA), 200 *μ*M deoxyribonucleotide triphosphates (dNTPs), 5 mM MgCl_2_, 10 pmol forward and reverse primers and 2 units GoTaq polymerase (Promega). PCR conditions were as follows: 95°C for 10 min, followed by 35 cycles at 94°C for 20 sec, 60°C for 30 sec and 72°C for 1 min, and later 3 min at 72°C. Aliquots of the PCR product were tested on 2% agarose gel. Shrimp alkaline phosphatase treatment of the PCR product, *in vitro* transcription to RNA and T-specific cleavage by RNAse A were performed according to the Sequenom protocol using a MassCLEAVE kit (Sequenom). The reaction product was robotically dispensed onto silicon chips for Sequenom mass spectrometry detection.

### RNA extraction and quantitative (q)PCR

RNA was extracted using TRIzol (Life Technologies, Carlsbad, CA, USA) according to the manufacturer’s instructions. For qPCR, 2 *μ*g RNA was reverse transcribed to cDNA according to the instructions of the High Capacity cDNA Reverse Transcription kit (Applied Biosystems, Foster City, CA, USA). TaqMan Real Time PCR (Life Technologies) was carried out with 0.2 *μ*g cDNA made up to a total reaction volume of 20 *μ*l with buffer, including ROX (Life Technologies), 0.2 mM dNTP, 5 mM MgCl_2_, 30 *μ*g bovine serum albumin, 5 pmol both forward and reverse primers, 5 pmol TaqMan probe (Life Technologies) and 0.5 units IMMOLASE (Bioline, Taunton, MA, USA). Primer sequences are shown in Table I. GAPDH was selected as a housekeeping gene for relative expression analysis. Samples were run on Real Time PCR 7500 (Applied Biosystems) at 95°C for 10 min, followed by 40 cycles at 95°C for 15 sec and 60°C for 1 min.

### Statistical analysis

CpG methylation was considered a continuous variable. Asymmetric distribution required non-parametric testing with Kruskal-Wallis one-way analysis and Mann-Whitney U tests. For qPCR, Student’s t-test was performed. qPCR results were analysed using the ΔΔCT method and compared with GAPDH. Correlations were calculated using Spearman’s rank correlation. Overall survival was calculated with Kaplan-Meier analysis and validated by log-rank tests. Statistical analysis was performed with GraphPad Prism 5 for Windows (GraphPad Software Inc., La Jolla, CA, USA). P<0.05 was considered to indicate a statistically significant difference.

## Results

### Tumor and control samples

A total of 69 samples were used: 43 GBM, 16 oligodendroglioma and 10 samples of non-tumor brain (excised from epilepsy patients). All samples were obtained at diagnosis and no relapse or secondary tumors were evaluated.

**Figure 2:**
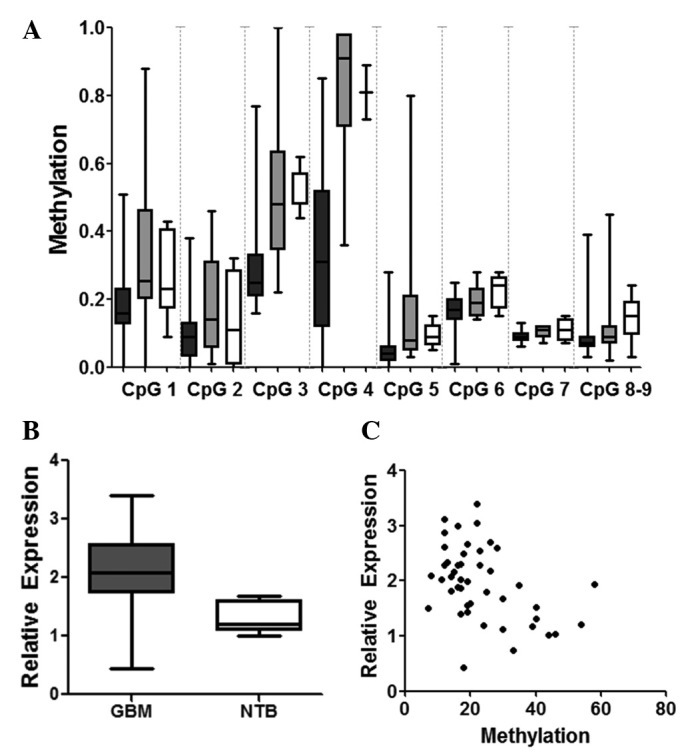
(A) Specific CpG-site RTVP-1 methylation in GBM (black), oligodendroglioma (grey) and NTB (white). P<0.001 for CpG 3, CpG 4, CpG 5 and CpG 6 (GBM vs. others). Bars represent median and interquartile range; whiskers represent range.. (B) RTVP-1 expression levels (quantitative polymerase chain reaction) in GBM compared with NTB sample (P<0.001). (C) Correlation between RTVP-1 methylation and expression. GBM, glioblastoma; NTB, non-tumor brain tissue; RTVP-1, related to testes-specific, vespid and pathogenesis protein-1.

RTVP-1 promoter methylation analysis using EpiDesigner software (Sequenom) revealed 9 CpG sites with 2 selected amplicons, covering 600 bp upstream and downstream of the RTVP-1 TSS (Fig. 1). Methylation analysis was performed on all 69 samples. Average RTVP-1 promoter methylation was significantly lower in GBM (15%) compared with non-tumor brain samples (25%; P=0.001). Specific CpG analysis revealed that all 9 CpG sites examined in the promoter area had lower methylation in GBM samples compared with controls (Fig. 2A). CpG 3 and CpG 4 (located −251 and −210 bp upstream of the RTVP-1 TSS, respectively) demonstrated the most significant methylation difference between sample groups (P<0.001). RTVP-1 promoter methylation in GBM was also significantly lower when compared with oligodendroglioma (P=0.001). RTVP-1 mRNA expression was significantly higher in GBM compared with the control group (median log expression with interquartile range, 2.06±0.69 in GBM vs. 1.33±0.26 in non-tumor brain; P<0.001); (Fig. 2B). RTVP-1 expression was inversely correlated with average promoter methylation (r=−0.4584; P=0.001; Fig. 2C).

### Promoter methylation status as a prognostic marker in GBM

The methylation pattern of RTVP-1 had great inter-sample variability in GBM compared with the narrow methylation ranges observed in oligodendroglioma samples and non-cancerous controls. Thus, several GBM samples were found to differ from the general malignant methylation pattern. The clinical significance of this pattern was assessed.

**Figure 3:**
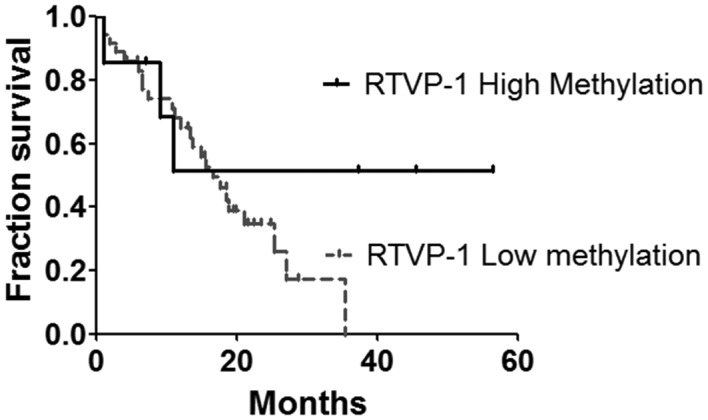
Kaplan-Meier plot to determine overall survival of glioblastoma patients based on RTVP-1 promoter methylation status: RTVP-1^high-meth^ is indicated by the black line and RTVP-1^low-meth^ by the grey dotted line. No statistically significant difference was identified. RTVP-1, related to testes-specific, vespid and pathogenesis protein-1.

The RTVP-1 promoter was hypermethylated in 7 GBM samples, similar to non-tumor samples. These samples, defined as RTVP-1^high-meth^, were compared with the 36 samples of hypomethylated RTVP-1 (RTVP-1^low-meth^). No significant differences were identified between RTVP-1^high-meth^ and RTVP-1^low-meth^ in basic characteristics, including median age (54 and 57 years, respectively), gender (57 and 55% in male patients, respectively) and tumor location. There was a trend towards increased overall survival in RTVP-1^high-meth^ patients verses RTVP-1^low-meth^ patients which did not reach statistical significance (Fig. 3).

## Discussion

In this study, the promoter methylation profile of RTVP-1 in GBM was evaluated. Compared with non-tumor brain, GBM samples were found to have hypomethylated RTVP-1 promoters.

RTVP-1 is normally expressed in the heart, spleen, muscle, bone marrow, placenta, adrenal and prostate (8). RTVP-1 behaves differently in various malignancies. In GBM, RTVP-1 is an overexpressed oncogene associated with increased proliferation, enhanced invasion and inhibition of apoptosis (6–9,15). By contrast, RTVP-1 is underexpressed in prostatic carcinoma, in which it is described as a tumor suppressor gene, acting via p53-dependent and -independent regulation (16). Furthermore, overexpression of RTVP-1 has been found to induce apoptosis in prostate cancer cell lines and *in vivo* models of prostate cancer (10,16–18). RTVP-1 expression in prostate cancer is downregulated epigenetically via methylation of the promoter (10). Promoter hypermethylation has also been found to reduce RTVP-1 expression in acute myeloid leukemia patients compared with lymphoblastic leukemia, chronic myeloid leukemia and remission bone marrow (11). Promoter hypomethylation with high RTVP-1 expression was identified in Wilms’ tumor (12). A similar correlation was recently described in melanoma (13).

To the best of our knowledge, this report is the first to identify similar promoter regulation in brain malignancies. Additionally, the expression of RTVP-1 was significantly higher in GBM compared with control non-tumor brain, in agreement with previous reports (6,7). An inverse correlation between RTVP-1 expression and promoter methylation was successfully demonstrated.

Recently, microRNA-137 (mir-137) was described as a regulator of RTVP-1 (15). Downregulation of mir-137 contributes to the high expression of RTVP-1 in glioblastoma. The current study describes further loss of regulatory control of RTVP-1 in GBM by promoter methylation. Unlike GBM, RTVP-1 was hypermethylated in oligodendroglioma, another astrocytic tumor. While this may indicate specificity of RTVP-1 hypomethylation in GBM, it may also reflect general hypermethylation observed in oligodendrogliomas (19,20), similar to the methylation pattern in secondary but not primary GBM (21).

The majority of known methylation-controlled promoters contain CpG-rich sites (CpG islands). The RTVP-1 promoter region contains a low number of CpG sites. Only 16 sites were identified within 1,000 bp upstream and downstream of the TSS. The method used for methylation analysis in the present study (Sequenom mass spectrometry detection of T-cleaved products of bisulfite-treated DNA) provided quantitative data through the ability to assess single-CpG methylation. This method has been used previously in similar studies and appears to have the benefit of being a sensitive quantitative method (14,15). This offers an advantage in CpG-poor regions, including the RTVP-1 promoter, and in identification of specific CpG sites, the methylation of which may significantly impact mRNA expression (22).

Finally, among glioblastoma samples, few were significantly hypermethylated, correlating with lower expression of RTVP-1, similar to non-tumor brain samples. In subjects with highly methylated RTVP-1, a trend towards prolonged survival was detected which did not reach statistical significance. Since RTVP-1 has oncogenic features in GBM, silencing via hypermethylation may have prognostic benefits. Further testing using additional samples are required to confirm this.

In conclusion, this study has shown for the first time that RTVP-1 is regulated by methylation in tissues of brain origin. In GBM, high expression of RTVP-1 is associated with promoter hypomethylation of this gene. GBM samples with hypermethylated RTVP-1 were associated with a tendency toward improved overall survival.

## Figures and Tables

**Table I:** PCR and qPCR primers.

Gene	Sense	Primer sequence, 5′-3′	Amplicon length, bp
PCR (C–T converted design^a^)			
RTVP-1			
Amplicon 3	Forward	TTTTATTTAATAGGTGGTTGAGGTT	424
	Reverse	CCAAAAAAAATTCTAAAATCTCCAAA	
Amplicon 4	Forward	TTGGAGATTATTATTTTTGGAGATTT	323
	Reverse	CCTTAAAAAACTACAATCCAAAACC	
qPCR			
RTVP-1	Forward	CAAGTGTTTGGACAATCTCTGTGTTA	80
	Reverse	GCCAGCCTGGATATACAACAGAGT	
	TaqMan probe	FAM-CCGACAGCGAGACCAAGTCAAACGT-BHQ-1	
GAPDH	Forward	CCTCCCGCTTCGCTCTCT	64
	Reverse	GGCGACGCAAAAGAAGATG	
	TaqMan probe	FAM-TCCTCCTGTTCGACAGTCAGCC-BHQ-1	

a As per the manufacturer’s instructions, all forward primers had a 5′-AGGAAGAGAG tag and all reverse primers had a 5′-CAGTAATACGACT CACTATAGGGAGAAGGCT tag.

PCR, polymerase chain reaction; qPCR, quantitative PCR; RTVP-1, related to testes-specific, vespid and pathogenesis protein-1.
